# Feasibility of Proton Transmission-Beam Stereotactic Ablative Radiotherapy versus Photon Stereotactic Ablative Radiotherapy for Lung Tumors: A Dosimetric and Feasibility Study

**DOI:** 10.1371/journal.pone.0098621

**Published:** 2014-06-02

**Authors:** Benjamin Mou, Chris J. Beltran, Sean S. Park, Kenneth R. Olivier, Keith M. Furutani

**Affiliations:** Department of Radiation Oncology, Mayo Clinic, Rochester, Minnesota, United States of America; Institut Gustave Roussy, France

## Abstract

Stereotactic ablative radiotherapy is being increasingly adopted in the treatment of lung tumors. The use of proton beam therapy can further reduce dose to normal structures. However, uncertainty exists in proton-based treatment plans, including range uncertainties, large sensitivity to position uncertainty, and calculation of dose deposition in heterogeneous areas. This study investigated the feasibility of proton transmission beams, i.e. without the Bragg peak, to treat lung tumors with stereotactic ablative radiotherapy. We compared three representative treatment plans using proton transmission beams versus conformal static-gantry photon beams. It was found that proton treatment plans using transmission beams passing through the patient were feasible and demonstrated lower dose to normal structures and markedly reduced treatment times than photon plans. This is the first study to demonstrate the feasibility of proton-based stereotactic ablative radiotherapy planning for lung tumors using proton transmission beams alone. Further research using this novel approach for proton-based planning is warranted.

## Introduction

Stereotactic ablative radiotherapy (SABR) plays an essential role in the treatment of patients with medically inoperable early stage lung cancer and oligometastasis. The use of protons for lung SABR is emerging as an appealing treatment option because of its potential to deliver higher doses of conformal radiotherapy and spare normal tissues better than traditional photons [1], [2], [3], [4]. This can be achieved because of the natural characteristics of proton beams that deposit its dose at depth with no exit dose, referred to as a Bragg peak. However, conventional dosimetric models fail to accurately model how protons scatter and deposit dose in highly heterogeneous areas which leads to uncertainties in proton treatment plans [5]. In addition, the uncertainties in the stopping power of the various tissues in the body and the interplay effect between spot scanning proton therapy and the target motion leads to large uncertainties in the treatment of lung tumors [5], [6].

In this study, we report on the feasibility of proton transmission-beam SABR (PT-SABR) for lung tumors which uses the transmission portion of a spot scanning proton beam, i.e. without the Bragg peak. This technique eliminates the major uncertainties of proton therapy mentioned above by having the proton beams pass through the patient. In addition, the use of the transmission beam allows an entire field to be treated in one breath hold. This quick treatment and decreased uncertainties lead to smaller planning volumes. To the best of the authors’ knowledge, this is the first report on the use of this novel approach to plan SABR with protons without using the Bragg peak, which may have dosimetric advantages over photon treatments.

## Materials and Methods

### Ethics Statement

Written informed consent was obtained from all patients registered in the SABR database. This study, including the consent procedure, was approved by the Mayo Clinic institutional review board.

### Patient Cohort

Patients were identified from a prospectively collected, institutional database of patients treated with SABR. Patients with lung tumors less than one centimetre in maximum dimension were included. The radiation treatment plans of three patients were extracted from the treatment planning system. All patients were treated using three-dimensional conformal multiple static-gantry photon beams. Plans were normalized so that 95% of the planning target volume (PTV) received at least 95% of the prescription dose. The prescription doses for these plans were adjusted to 34 Gy in one fraction based on the recently reported results of Radiation Therapy Oncology Group (RTOG) 0915 which established this dose fractionation regimen as a possible standard dose to be used in future trials [7]. Dose calculations for photon plans used the anisotropic analytical algorithm.

### Proton Treatment Planning

A machine was commissioned in Eclipse v.10 (Varian Medical Systems, Palo Alto, CA) which allowed for planning and calculating transmission dose plans. The spot size (sigma) of the transmission beam, which had an energy of 229 MeV, was 2.2 mm. A proton plan that only used the transmission portion of the beam was created for each patient. Proton beam arrangements were selected so that no beams entered through the heart or spinal cord and allowed up to two non-coplanar beams. Four to five beams were used to keep the skin dose comparable to photon plans. The energy of the protons for each spot of a field was 229 MeV; this ensured the Bragg peak was not located within the patient. Dose calculations for the transmission portion of the proton beam were verified with Monte Carlo (Geant4). The proton plans were normalized so that the internal target volume (ITV) receives at least 95% of prescription dose including when range and position errors were included (3.5% and 2 mm), which is standard for spot scanning proton therapy. ITVs were created based on motion of the gross tumor volume in three dimensions using four-dimensional computed tomography image data. The dose constraints from RTOG 0915 were compared for the photon and proton plans as well as the total time that would be required to deliver the treatment. The radiotherapy delivery time per beam was estimated at 1 nC per second for proton therapy, which is readily achievable by most spot scanning proton centers, and 600 MU per minute for the photon plans. Differences in dosimetric and treatment planning parameters between photon and proton plans were analyzed with two-sided paired t-tests using SAS version 9.2 (SAS Institute Inc., Cary, NC).

## Results

**Figure 1:**
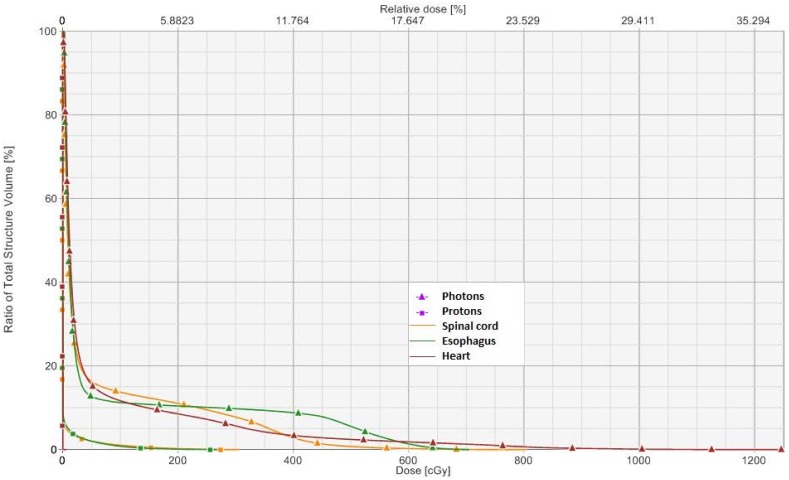
Dose-volume histogram comparison of organs at risk.

The ITVs of the three tumors measured 0.22, 0.42, and 0.99 cubic centimeters. All three proton plans had excellent coverage of the ITV. For all ITVs, over 99.4% of the volume received at least 95% of the prescription dose, including when uncertainties were examined. This was comparable with the photon plans where 100% of the ITVs received at least 95% of the prescription dose. For most normal tissues, lower doses to these organs were achieved with the proton plans compared to the photon plans (Table 1). In fact, (near) complete sparing of the spinal cord, heart, and esophagus was possible with protons through careful selection of beam angles (Figure 1).

**Table 1:** Dosimetric comparison of photon and proton plans.

Parameter		Photon		Proton		P-value
		Mean	Range	Mean	Range	
Internal target volume (cc)		0.54	0.22–0.99	0.54	0.22–0.99	N/A
Spinal cord						
	Maximum dose (Gy)	5.66	2.39–8.07	1.97	0.00–3.06	0.04
Lungs (bilateral)						
	Mean lung dose (Gy)	1.35	0.95–1.92	0.69	0.03–1.36	0.12
	V20 (%)	0.66	0.39–1.20	0.49	0.16–1.01	0.06
	V5 (%)	7.32	5.4–11.30	6.65	2.96–11.70	0.56
Heart						
	Mean dose (Gy)	8.36	6.27–12.51	0.00	0.00–0.00	0.13
Skin						
	Maximum dose (Gy)	11.75	9.86–13.28	11.40	7.37–16.23	0.89
Esophagus						
	Maximum dose (Gy)	6.49	2.98–9.43	3.40	0.00–7.51	0.05
Homogeneity Index		1.25	1.21–1.29	1.07	1.03–1.11	0.06
Conformity Index		17.14	8.23–30.05	3.47	2.17–4.64	0.15

**Figure 2:**
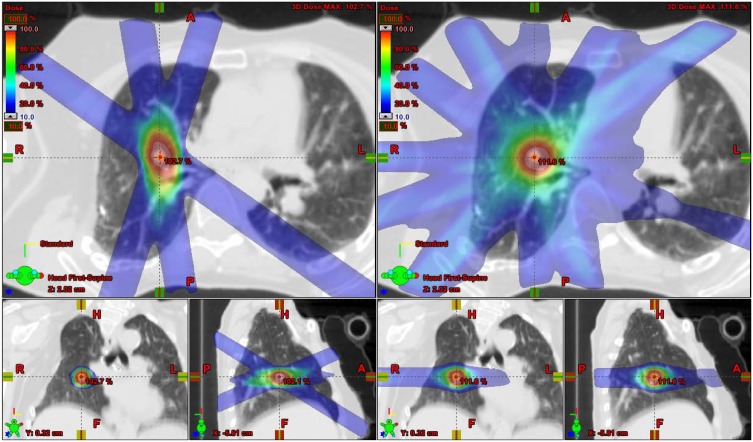
Comparison of isodose distributions. Proton (left) and photon (right) treatment plans.

Proton plans used four to five non-coplanar beams compared to nine to ten beams for photon plans (Figure 2). The average number of monitor units per field was 818 (range 758–871) with photons and only 38 (range 31–59) with protons. This would translate to an average beam-on time per field of 82 seconds versus 6 seconds for photon and proton plans, respectively. These differences in monitor units and beam-on time were statistically significant with P<0.01(Table 2).

**Table 2:** Comparison of treatment time between photon and proton plans.

Parameter	Photon		Proton		P-value
	Mean	Range	Mean	Range	
Total monitor units (MU)	7929	6820–8713	178	122–235	<0.01
Fields	9.7	9–10	4.7	4–5	N/A
Average MU/field	818	758–871	38	30.5–46.9	<0.01
Beam on time per field (seconds)	81.8	75.6–87.1	5.8	4.7–7.2	<0.01

## Discussion

Exploiting the transmission beam in proton therapy planning has significant potentials for dose escalation and re-irradiation in lung tumors and eliminates the concern over the uncertainty of the stopping power and its impact on the Bragg peak location. PT-SABR planning requires fewer beams than photons, and careful selection of optimal beam angles allows for minimal dose to adjacent normal tissues and tumor dose escalation which may translate to improved local control rates. RTOG 0915 showed that 34 Gy in a single fraction was comparable to 48 Gy in four fractions [7], and the dosimetric constraints from the protocol were easily achieved using both proton and photon plans for patients in this study. Further optimization with proton therapy can allow even higher doses to be delivered while still respecting established dosimetric constraints for normal tissues. This may translate to better tumor control but requires more investigation in a clinical setting.

Patients planned with PT-SABR required fewer beams (5 vs. 10) which reduce the total treatment time and the low dose outside the tumor. The average monitor units per field for PT-SABR plans were a fraction of those needed for the photon plans (Table 2). This translates to a beam on time per field of between 5 and 10 seconds for the PT-SABR plans compared to 75 to 90 seconds for photon plan. This 5 to 10 second time estimate is based on a conservative 1 nC/sec dose rate, however, new proton centers may be able to achieve greater than 2 nC/sec, thereby reducing this time by a factor of 2. By decreasing the treatment time to less than 10 seconds per field, breath-hold techniques may be better tolerated in greater number of lung cancer patients with suboptimal lung function. Breath-hold technique would minimize tumor motion (i.e. ITV) leading to a smaller overall irradiation volume, and interplay would not be a significant issue [8]. Spot scanning proton therapy that utilizes the Bragg peak would require a larger planning volume due to the various uncertainties that need to be taken into account; and it would require a longer treatment time due to the use of multiple proton energies. Each change in energy requires several seconds (2 to 7) and at least 5 to 10 energies would be required for these treatments. Volumetric modulated arc therapy (VMAT) with photons may decrease treatment times compared to multiple static-gantry beams. However, VMAT comes at the cost of larger volumes of normal tissue receiving low doses of radiation since the beam is continuously on as it rotates about the patient. The use of four to five proton transmission beams achieves both shorter treatment times as well as a lower integral dose to the body.

The dosimetric data of the normal tissues in the photons plans met the constraints of RTOG 0915. The dosimetric gains of protons over these plans may be considered modest and the statistical analysis comparing plans is limited by the small sample size. However, in some plans, the dose to particular critical organs can be avoided completely without compromising target coverage by choosing beam arrangements appropriately. This may be beneficial in treating patients with tumors in challenging locations [9] or recurrent tumors that have had prior radiotherapy. The interim analysis of RTOG 0617 reported local failure rates of 25% and 34% in the standard and high dose RT arms [10], and therefore, re-irradiation may play a role in this subset of patients who fail after definitive chemoradiotherapy. For these patients, keeping dose at or near zero to the spinal cord, heart, lungs, or other critical structures is feasible with protons.

Planning with PT-SABR using only transmission beams without the Bragg peak is feasible. This proof of principle as described in our study eliminates the uncertainty of proton dose distribution in lung tumors which has the potential to underdose the target and overdose surrounding normal tissues. Proton therapy planning with this technique also demonstrates better sparing of normal tissues and fast treatment times than photon plans. Further study of this novel approach to proton SABR is warranted.
